# Review of *As Nomadism Ends: The Israeli Bedouin of the Negev* by Avinoam Meir

**DOI:** 10.1186/s13570-021-00208-2

**Published:** 2022-01-10

**Authors:** Steven C. Dinero

**Affiliations:** Rawlins, WY 82301 USA

**Keywords:** Palestine, Jewish state, Tribe, Sedentarization, Planned towns

## Abstract

Meir, Avinoam

*As Nomadism Ends: The Israeli Bedouin of the Negev*

London & New York: Routledge, 2020

268 pages, ISBN: 978-0-3671-6054-8 $49.95 (paper).

It is hardly uncommon to publish a second or third edition of a volume that has proven to be particularly popular. Often, a new edition might appear with a revised preface or introduction, allowing the author to reflect back upon some of the main concepts and ideas that underpin his or her argument and to consider, years hence, the degree to which such views still hold credence. Such exercises make a considerable contribution to the literature especially when—if a scholar is honest and forthright—some of the views once expressed turn out to have been proven wide of the mark.

But then, isn’t that the whole point of scientific inquiry? Those of us who have entered the academy over the years are not soothsayers after all but, rather, are just educated folks who, given hours spent in the library and in the field, have sought to tell the world a small part of what is, after all, a very big story of what makes this universe of ours tick. Being “right” is surely the hopeful outcome for most of us; doing our best with the information we have at the moment, however, is probably the most that we can really hope for.

This brings me to the present monograph under review. The author, Dr. Avinoam Meir, now Professor Emeritus at Ben Gurion University of the Negev, has been a friend and colleague for over a quarter-century. The copyright on the volume under discussion states that this version is a product of the early twenty-first century, 2020 to be exact. But the truth of the matter is that this is in fact a reprint of a volume that was researched throughout the 1980s to 1990s and was published in 1997 with a different press (Westview). I own the original and consulted it regularly when it first came out. I compared the two and found that there is no new preface, introduction or any other updates in this new printing. Even the “About the Author” blurb is identical.

That said, my copy has sat on my bookshelf for some 20-plus years, and I haven’t consulted it in some time. As such, I sought to read the new version with new eyes. At my age, it is fair to say that a lifetime has passed since I last read the book in its entirety. I strived not to approach it in terms of 20-20 hindsight; none of us are clairvoyant after all. Still, this is the “2020” version and so I tried to view it as such.

From the outset, Meir is clear that, as the title of the volume suggests, nomadism may be found within a continuum of evolutionary social and economic development. This lifestyle, he offers, surely had a purpose at one time in human history but, as he seeks to illustrate throughout the volume, that era is rapidly coming to an end. Nomadism is “ending”, he seeks to reveal here, because it is being rendered obsolete; most certainly, it had its purpose and value at one time but that time is over. As he writes in the volume’s early pages “the major focus of the present book is the processes that occurs as nomadism ends rather than those causing the end of nomadism” (2). Put differently nomadism, be it in the Negev or elsewhere, is not ending due to some sort of external attack but rather, it is happening *naturally* because it is, for all intents and purposes, an anachronism.

The modernist assumption that underpins this view is at the crux of much of the argument which then runs through the narrative of the rest of the text. Certain basic provisos provide the superstructure for Meir’s primary argument. In Chapter 2, for example, he outlines the theoretical basis for the rest of the volume. Here, his primary argument rests upon the presumably necessary shift taking place among more “traditional” nomadic communities as they transform from a more “tribal” orientation to that of a more “individualized” orientation. He strives to point out that such “notions of autonomy and privacy leads to the idea of self-development” (13), that is, to a desire to work towards one’s own success and growth as opposed to (and at the expense of) the “success of the group”.

Such an argument certainly holds some truth. More to the point, such shifts serve as one of the key foundations of late-stage capitalist growth inherent in our ever-expanding neoliberal global economy as well—but for the moment, I’ll opt to leave that issue aside.

As for the issue of living in dispersed clusters rather than concentrated urban environments, Meir offers that here too, sedentarization into highly agglomerated spaces is proving to be a preferred choice among many formerly nomadic tribes. And with it comes shifts with significant ramifications. Over several pages, he contends that along with this process has evolved the idea of *territoriality*—perhaps best viewed in terms of the privatization of land. Additional changes in women’s roles (i.e. women may now enter the workplace), in fertility patterns (i.e. smaller family size) and in the role of children (i.e. fewer children who are not viewed as labourers but rather, who are able to access formal education, male and female alike) have been soon to follow. The end result, he contends, has been a number of improvements in “communal well-being” overall as social resources once irrationally distributed by the tribe are now provided rationally through modern systems and agencies (35).

Subsequent chapters then address these issues in more detail. Chapter 3 provides a brief overview of “The Jewish-Bedouin Encounter” in Palestine’s Negev (Naqab) Desert in its earliest years. Chapters 4, 5, 6 and 7 continue the discussion. Much of this story is well-known by now, having been retold numerous times over the years. To summarize, the State sought to control and encapsulate the Bedouin community, in part for political reasons but also in the hopes of providing badly needed systems to facilitate development. They believed that (as Meir explains here at length) by forcing the community into small planned towns with various amenities, utilities and other services, they could forcibly “modernize” the Bedouin and, at the same time, get them to forget who and what they were—Muslim Arab Palestinians who had a connection to the land that they had lived on for generations (not to mention the social connections they shared with other Palestinian Arabs in the region). And yet, as events on the ground prove today, they couldn’t have been more wrong.

In that regard, Chapter 8, “The Political Dimension: The Bedouin and the State”, still resonates decades after it was written. Social and economic challenges are rife, including unemployment, violence, conflict with neighbouring Jewish communities, political protest, educational difficulties including high dropout rates, gang activity—the list goes on. Despite (because of?) questionable planning practices, circumstances in the Bedouin sector remain problematic at best, a smouldering powder keg of anger and resentment.

Given the age of this text, it is not surprising to find a number of examples where Meir turned out, in fact, to be “wide of the mark”. As but one example, he postulates that the Bedouin community is “too patriarchal” to accommodate “modern ideas concerning the status of women” (223). As it happens, Bedouin women today have moved quickly into all realms of work, education and beyond. But more to the point, the foundational aspects of “traditional” nomadic life that he considers as relicts of the past—living in small, socially distant clusters; working from home; deterritorialization (facilitated by the ubiquity of various digital technologies); raising/growing one’s own food—have now become the centerpieces of twenty-first century life, suggesting that perhaps groups like the Negev Bedouin were in fact ahead of their time.

Indeed as communities across Europe, the USA, and beyond begin to socially and economically reconfigure in the post-COVID-19 pandemic era, it seems all but certain that the idealization of the high rise-dominated urban agglomeration may soon become passé. Most certainly, Dr. Meir could never have anticipated any of this in 1997. Still, the text stands as a significant contribution to the literature given the time period when it was originally published.

That said, perhaps we should all be humbled by recent events and realize that we academics ought to tread lightly when we make certain bold assumptions as someday, our words may come back to us in unexpected ways.

Or in this case, to paraphrase Mark Twain’s now well-worn words, “The reports of the death of nomadism have been greatly exaggerated”.

## Data Availability

None.

